# Development and validation of a nomogram for the early prediction of drug resistance in children with epilepsy

**DOI:** 10.3389/fped.2022.905177

**Published:** 2022-08-30

**Authors:** Hua Geng, Xuqin Chen

**Affiliations:** Neurology Department, Children's Hospital of Soochow University, Suzhou, China

**Keywords:** drug-resistant epilepsy, prediction model, nomogram, drug-resistant epilepsy (DRE), children, drug-sensitive epilepsy (DSE)

## Abstract

**Background and purpose:**

This study aimed to effectively identify children with drug-resistant epilepsy (DRE) in the early stage of epilepsy, and take personalized interventions, to improve patients' prognosis, reduce serious comorbidity, and save social resources. Herein, we developed and validated a nomogram prediction model for children with DRE.

**Methods:**

The training set was patients with epilepsy who visited the Children's Hospital of Soochow University (Suzhou Industrial Park, Jiangsu Province, China) between January 2015 and December 2017. The independent risk factors for DRE were screened by univariate and multivariate logistic regression analyses using SPSS21 software. The nomogram was designed according to the regression coefficient. The nomogram was validated in the training and validation sets. Internal validation was conducted using bootstrapping analyses. We also externally validated this instrument in patients with epilepsy from the Children's Hospital of Soochow University (Gusu District, Jiangsu Province, China) and Yancheng Maternal and Child Health Hospital between January 2018 and December 2018. The nomogram's performance was assessed by concordance (C-index), calibration curves, as well as GiViTI calibration belts.

**Results:**

Multivariate logistic regression analysis of 679 children with epilepsy from the Children's Hospital of Soochow University (Suzhou Industrial Park, Jiangsu Province, China) showed that onset age<1, status epilepticus (SE), focal seizure, > 20 pre-treatment seizures, clear etiology (caused by genetic, structural, metabolic, or infectious), development and epileptic encephalopathy (DEE), and neurological abnormalities were all independent risk factors for DRE. The AUC of 0.92 for the training set compared to that of 0.91 for the validation set suggested a good discrimination ability of the prediction model. The C-index was 0.92 and 0.91 in the training and validation sets. Additionally, both good calibration curves and GiViTI calibration belts (*P*-value: 0.849 and 0.291, respectively) demonstrated that the predicted risks had strong consistency with the observed outcomes, suggesting that the prediction model in both groups was perfectly calibrated.

**Conclusion:**

A nomogram prediction model for DRE was developed, with good discrimination and calibration in the training set and the validation set. Furthermore, the model demonstrated great accuracy, consistency, and prediction ability. Therefore, the nomogram prediction model can aid in the timely identification of DRE in children.

## Introduction

Epilepsy is a disorder of the brain with the characteristic of an enduring predisposition to generate epileptic seizures, which has been recognized as one of the most prevalent neurological and psychiatric conditions by the World Health Organization (WHO). Currently, there have been more than 70 million patients with epilepsy worldwide (1), and its prevalence usually peaks among those <1 year old or > 50 years old (2). Three generations of antiepileptic drugs have been developed. Although AED therapy has played an effective role in stopping seizures in a great number of patients with epilepsy, and seizure control remains unsuccessful for some of them. As reported in studies, 20–30% become resistant to treatment with AEDs (3–5). Frequent seizures would cause neuron loss, axon germination, glial hyperplasia, and neural network remodeling (6), which could be fatal due to either the direct outcomes of seizures (such as sudden death from epilepsy, long falls, etc.) or the indirect outcomes of seizures (such as inhalation pneumonia, suicide, etc.) (7). Seizures seriously affect the development of the nervous system, resulting in growth retardation, low cognitive function, and a series of neuropsychological problems. The mortality rates of drug-resistant epilepsy (DRE) are four to seven times higher than those of patients with drug-sensitive epilepsy (DSE) (8). Kwan et al. indicated that some patients with DRE are possible to be identified early, and thus can be targeted for rational therapies or surgeries (9). Notably, the early risk factors for DRE, when paired with the prediction model, may offer a basis for personalized treatment. However, the early risk factors for DRE are still debatable, and there is no effective tool for diagnosing DRE. Therefore, it is vital to develop an early prediction model of DRE that will not only offer a basis for early DRE diagnosis and treatment selection but will also increase doctor–patient communication and achieve optimal allocation of healthcare resources. Most of the existing prediction models have focused on identifying independent risk factors for DRE (10). Scholars have developed several tools, including formulas, machine learning methods, prediction models of logistic regression, clinical prediction rules (CPR), deep learning methods, molecular diagnosis model, and integrative prediction algorithm based on combined clinical-EEG functional connectivity features and circulating microRNAs from plasma small extracellular vesicles, for the early prediction of the probability of DRE (11–18). Nevertheless, all of these tools were neither simple enough nor convenient for practical application. Nomogram is an intuitive prediction model which is graphically presented. Each independent risk factor listed in the nomogram can be quantified. Benefiting from these advantages, nomogram has been widely used in the diagnosis and prognosis of diseases. Thus, our team decided to develop a nomogram prediction model for DRE. Every potential risk factor in the nomogram model would be analyzed and quantified, to individually predict the likelihood of DRE in children. Meanwhile, we would assess the discrimination and calibration of the nomogram prediction model in internal and external children with DRE, to provide a reliable tool that could be directly used in clinical practice.

## Materials and methods

### Patients

The predictive nomogram model was developed based on a retrospective study of children with epilepsy who visited the Children's Hospital of Soochow University (Suzhou Industrial Park, Jiangsu Province, China) between January 2015 and December 2017. Internal validation was conducted using bootstrapping analyses with 1,000 resamples. The external validation set comprised patients with epilepsy from the Children's Hospital of Soochow University (Gusu District, Jiangsu Province, China) and the Yancheng Maternal and Child Health Hospital between January 2018 and December 2018. All the patients were regularly followed up in the Department of Neurology for more than 36 months.

### Inclusion criteria

Currently, according to the guideline of the International League against Epilepsy (ILAE) 2010 (19), DRE is defined as a failure of adequate trials of two tolerated, appropriately chosen, and used antiepileptic drug schedules (whether administered alone or in combination) to achieve sustained freedom from seizure for three times the longest interval between seizures before the intervention (identified from seizures happened during the past 12 months), or 12 months. Thus, the inclusion criteria of DSE were as follows: (1) two unprovoked epileptic seizures more than 24 h apart; (2) sustained freedom from seizure for three times the longest interval between seizures before the intervention (identified from seizures happened during the past 12 months), or 12 months.

All the patients in the training set and validation set must regularly be followed up in the Department of Neurology more than 36 months after diagnosis.

### Exclusion criteria

Exclusion criteria for DRE were: (1) incomplete information: children with incomplete clinical and follow-up information; (2) diagnostic mistakes: children mistakenly diagnosed as epilepsy, such as vasovagal syncope, migraine, transient ischemic attack, cardiogenic attack, and arrhythmia; (3) treatment errors: children failed to receive AEDs corresponding to their types of epilepsy, with inappropriate pharmacokinetic effects; (4) dose errors: for AEDs whose blood concentration can be measured, their doses were too low, that is, blood concentration did not reach the therapeutic level, or the AED could not be tolerated by children; for AEDs whose blood concentration cannot be measured, their doses did not reach the minimum maintenance dose recommended by the International League Against Epilepsy (ILAE) (20) or the instructions of the manufactures; (Supplementary Table 2); (5) inducing factors of epilepsy: poor medication compliance; (6) other serious systemic diseases: such as severe liver and kidney diseases, or congenital heart disease.

Exclusion criteria for DSE were: (1) poor treatment compliance; (2) acute symptomatic attacks; (3) missing information: incomplete clinical and follow-up data; (4) severe systemic diseases.

### Data collection

According to the process, we collected the electronic medical histories of children with epilepsy in the past. Missing information was retrieved by phone call or directly contacting the children's guardians. The data in the questionnaire were systematically sorted and summarized. The personnel who were responsible for the collection and data input of medical history were trained by “Galaxy Plan” (which was a capacity-building project for professional doctors in epilepsy, held by the Chinese Anti-epilepsy Association), and obtained qualification certification, to ensure the accuracy and authenticity of data collection. Clinical history was obtained only from the child's legal guardian or long-term caregiver. Moreover, the original medical records and auxiliary examinations, as well as electronic medical records, were inquired for information validation, to ensure the authenticity and integrity of the data and to minimize recall bias. The information on explanatory variables were collected and documented at the time of the initial presentation of the patients to the study hospital. All the patients in the training set were regularly followed up in the Department of Neurology for more than 48 months after diagnosis. All patients were followed up for at least 36 months to guarantee sufficient time for the observation of therapeutic effect, disease progression, and outcome. This study complied with the principles of the Declaration of Helsinki, as well as its amendments, and was approved by the ethical committees of Children's Hospital of Soochow University and Yancheng Maternal and Child Health Hospital.

### Definition of variables

1. Gender: male or female.
2. Onset age: within 1 year of birth or more than 1 year old.
3. Status epilepticus (SE): with or without status epilepticus.
4. Focal seizure: The children with DRE in the research were categorized and grouped into three categories: focal seizure, generalized seizure, and unclassified seizure, based on the ILAE classification of epilepsy in 2017, as well as the symptoms of seizures and EEG results.
5. Clustered seizures: two or more seizures within 24 h.
6. Pre-treatment seizures: it was defined as the number of seizures before the treatment and was divided into > 20 and ≤ 20 times (clustered seizures in one day means one seizure).
7. Radiological abnormalities: According to ***MRI in Epilepsy*** by Horst Urbach (21), head MRI was divided into normal conditions and abnormal ones related to epilepsy. The head MRI abnormalities included: hippocampal sclerosis, epilepsy-related tumor, cortical malformations, neurocutaneous syndrome, traumatic change, vascular malformation, ischemic changes, infectious and inflammatory changes, and metabolic changes.
8. Etiology: According to ILAE's suggestion, the etiology of DRE was divided into six types, namely, genetic, structural, metabolic, immune, infectious, and unknown ones (22).
9. Development and epileptic encephalopathy (DEE) include three electroclinical entities: developmental encephalopathy (DE: in conditions where the cognitive development and behavior are impaired independently of the epilepsy onset), epileptic encephalopathy (EE: it is a progressive brain dysfunction caused by frequent epileptic seizures and/or epileptic discharge), as well as the combination of the two (DEE). In clinical practice, it was difficult to distinguish DE from patients with EE, thus patients with DE, EE, or both were included in this study and recognized as patients with DEE (22–24).
10. Initial EEG: Using a Nihon Kohden EEG-1200C (Tokyo, Japan) or Nicolet EEG-V36 (USA), the EEG electrodes were placed on the scalp, recording voltage potentials resulting from current flow in and around neurons, according to the International 10–20 system. The video EEG of the patient was monitored and recorded for no less than 4 h. According to ***Clinical*** ***Electroencephalography*** by Xiao-yan Liu (25), pediatric EEG could be divided into normal and abnormal ones. EEG abnormalities included inter-seizure abnormalities, abnormalities during seizures, and background abnormalities. The video EEG before the treatment was called the initial EEG.
11. Neurological conditions: Physical examinations of the neurological system would be conducted for all the children. The results would be divided into normal and abnormal ones. Neurological abnormalities included abnormalities in motor function, sensory function, nerve reflex, skin, and skull.
12. Perinatal asphyxia: with or without perinatal asphyxia. The definition of perinatal asphyxia in China provided by the neonatal committee of the Chinese Medical Doctor Association (20) was as follows: (1) Prenatal high-risk factors that may lead to asphyxia; (2) Apgar score ≤7 in 1 or 5 min, without spontaneous respiration established. (3) PH < 7.15 in the umbilical arterial blood sample; (4) other causes of low Apgar score were excluded.
13. History of febrile convulsion: with or without history of febrile convulsion. Patients who have ever had febrile convulsion for once were defined to have a history of febrile convulsion.
14. Family history: with or without a family history. Family history refers to epilepsy in a first-degree relative or sibling.
15. Multiple seizure forms: according to the ILAE classification (22), seizure forms ≥2 were defined as multiple seizure forms.

### Statistical analysis

Statistical analysis was performed by SPSS 21.0. The age and onset age in this study were not normally distributed, and thus were described as the median (interquartile interval). The categorical data were described as frequency (percentage). The continuous variables between groups were compared by the Mann–Whitney U test, and categorical variables were compared by the Chi-square (χ^2^) test. The risk factors were analyzed by univariate and multivariate logistic regression. The risk factors with *P* < 0.05 in the univariate analysis were deemed to indicate statistical significance, selected into the multivariate logistic regression analysis, and further screened by a stepwise forward method. Variance inflation factor (VIF), tolerance, and eigenvalue conditional indexes were used to detect collinearity.

A nomogram prediction model of individual DRE was developed with the independent risk factors of the multivariate regression analysis, using the rms, foreign, and gglot2 packages in R software. The corresponding scores for each predicting factor were listed in the nomogram, and then the score for all variables was summed up, mapping to a scale of outcomes that corresponded to the likelihood of DRE.

We tested the nomogram's performance through discrimination along with calibration. The nomogram was validated in the training and validation sets. The nomogram for DRE was subjected to 1,000 bootstrap resamples for internal validation to assess their predictive accuracies. The discrimination of the model was assessed through the receiver operating characteristic (ROC) curve, which was drawn using R software. A two-tailed P<0.05 was considered to be statistically significant. The nomogram's predictive accuracy was assessed using concordance (C-index), calibration curves along with GiViTI calibration belts. C-index's value is related to the predictive accuracy rate. The larger the C-index is, the higher the predictive accuracy rate would be. In addition, calibration plots of predictive DRE risk were assessed in the training and external validation sets. A perfect prediction would correspond to the 45° dashed line. The closer to the 45° dashed line, the predicted calibration curve is the better the predictive ability of the nomogram would be. GiViTI has been proposed as a graphical tool to identify ranges of probability where a model based on dichotomous outcomes mis-calibrates and applied to determine the goodness-of-fit of the prediction model (26). It has a calibration curve and a confidence belt. The calibration curve interprets the link of predicted risk with the observed outcome for various levels of risk, whereas the curve's confidence belt estimates the degree of uncertainty based on the true location of the curve. In the GiViTI calibration belt, after fitting a polynomial logistic function to the logit transformation of the predicted probability and outcome, the relationship between the predicted and observed outcomes is calculated. Through the calibration belt, 80% CI (marked in light gray) along with 95% CI (marked in dark gray) around the calibration curve are calculated. There would be a deviation from the bisector vector (dashed line) with statistical significance if the 95% CI does not cross the bisector (27).

## Results

### Baseline characteristics

**Figure 1:**
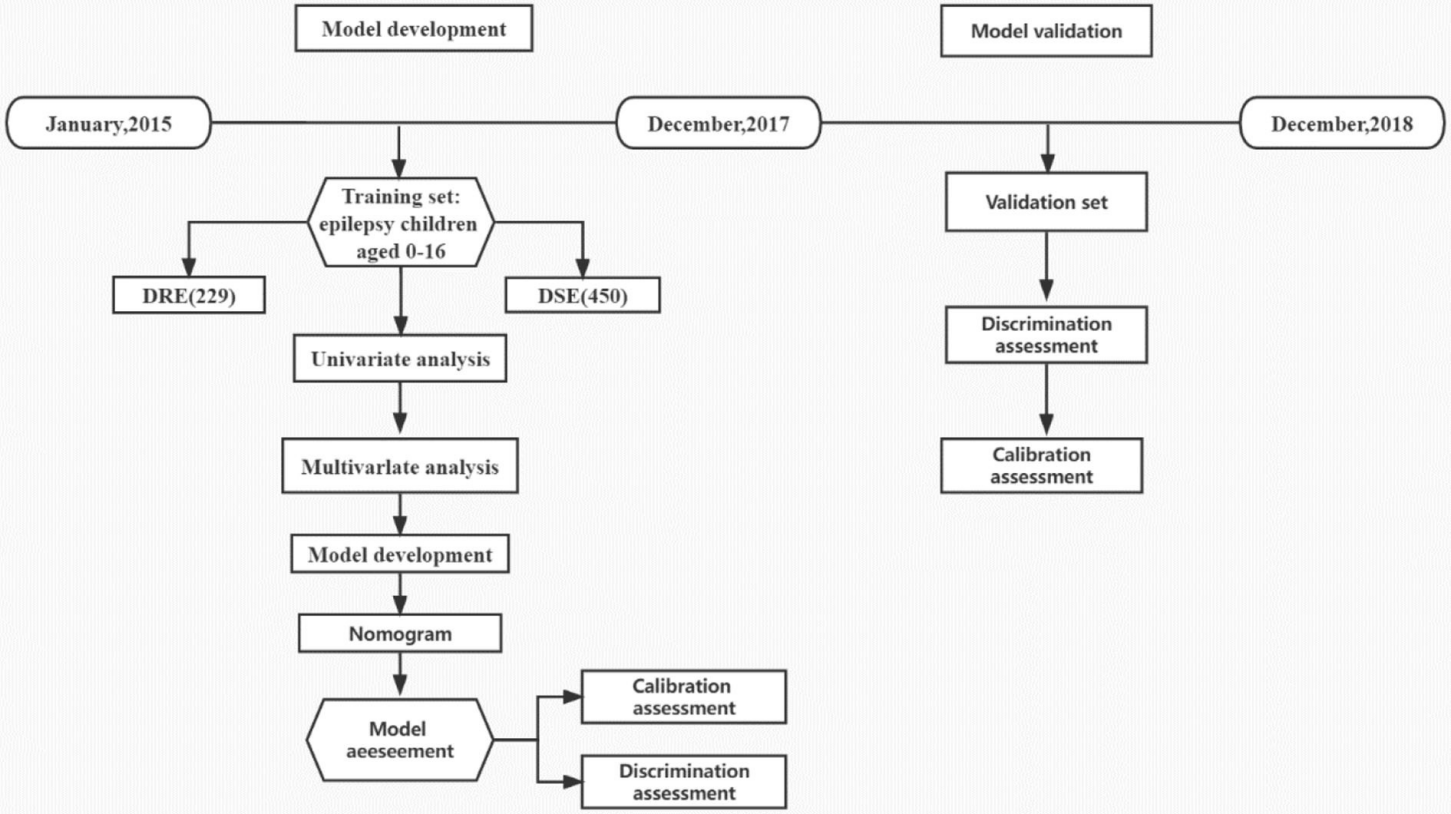
Technology roadmap.

A total of 856 children with epilepsy, who visited the Children's Hospital of Soochow University (Suzhou Industrial Park, Jiangsu Province, China) from January 2015 to December 2017, were evaluated, among which 177 children were excluded according to the inclusion criteria and exclusion criteria. Of the remaining 679 cases that were included in the training set for model development (407, 170, and 102 in preschool, school-age, and adolescence period, respectively), 229 belonged to the DRE group, and 450 were in the DSE group. Furthermore, there were a total of 347 patients included for external validation, including 189 children with epilepsy (64 in the DRE group, and 125 in the DSE group) who visited the Children's Hospital of Soochow University (Gusu District, Jiangsu Province, China) and 158 children with epilepsy (54 in the DRE group, and 104 in the DSE group) who visited Yancheng Maternal and Child Health Hospital from January 2018 to December 2018 (Figure 1). All the patients in the external validation set were regularly followed up in the Department of Neurology for at least 36 months from diagnosis of epilepsy. Patients with DSE in the external set were effectively controlled and their antiepileptic drugs were gradually withdrawn without recurrence. Finally, 1,026 children in total were included in this study, among which 679 children were in the training set, with 369 male (54.3%) and 310 female (45.7%), while 347 children were in the external validation set, with 200 male (57.6%) and 147 female (42.4%). There was no significant difference between the baseline characteristics of the two sets (Table 1). All the patients in the training set were regularly followed up in the Department of Neurology for more than 48 months after diagnosis, with the median follow-time reaching up to 66 months (range 48–84 months, interquartile range [IQR] 55.2–76.8 months), which was longer than the median of 42 months in the validation set (range, 36–48 months, IQR 38.4–45.6 months).

**Table 1:** Baseline characteristics of the training set and validation set.

	**Training set** **(*n =* 679)**	**Validation set** **(*n =* 347)**	**z/χ^2^**	***P* value**
Gender (%)	–	–	1.01	0.315
Male	369 (54.3%)	200 (57.6%)	–	–
Female	310 (45.7%)	147 (42.4%)	–	–
Onset age <1 (%)	–	–	1.47	0.225
Yes	153 (22.5%)	90 (25.9%)	–	–
No	526 (77.5%)	257 (74.1%)	–	–
SE (%)	–	–	1.28	0.258
Yes	74 (10.9%)	30 (8.6%)	–	–
No	605 (89.1%)	317 (91.4%)	–	–
Focal seizure (%)	–	–	0.15	0.696
Yes	359 (52.9%)	179 (51.6%)	–	–
No	320 (47.1%)	168 (48.4%)	–	–
Clustered seizures (%)	–	–	0.22	0.642
Yes	296 (43.6%)	146 (42.1%)	–	–
No	383 (56.4%)	201 (57.9%)	–	–
>20 pretreatment seizures (%)	–	–	0.01	0.919
Yes	260 (38.3%)	134 (38.6%)	–	–
No	419 (61.7%)	213 (61.4%)	–	–
Radiographic tests (%)	–	–	3.45	0.063
Abnormal	165 (24.3%)	103 (29.7%)	–	–
Normal	514 (75.7%)	244 (70.3%)	–	–
Etiology (%)	–	–	5.97	0.309
Genetic	78 (11.5%)	37 (10.7%)	–	–
Structural	77 (11.3%)	51 (14.7%)	–	–
Metabolic	31 (4.6%)	18 (5.2%)	–	–
Immune	19 (2.8%)	16 (4.6%)	–	–
Infectious	113 (16.6%)	48 (13.8%)	–	–
Unknown	361 (53.2%)	177 (51.0%)	–	–
DEE (%)	–	–	0.53	0.465
Yes	183 (27.0%)	101 (29.1%)	–	–
No	496 (73.0%)	246 (70.9%)	–	–
Initial EEG (%)	–	–	1.71	0.191
Normal	82 (12.1%)	52 (15.0%)	–	–
Abnormal	597 (87.9%)	295 (85.0%)	–	–
Perinatal asphyxia (%)	–	–	0.28	0.598
Yes	56 (8.2%)	32 (9.2%)	–	–
No	623 (91.8%)	315 (90.8%)	–	–
History of febrile convulsion (%)	–	–	3.23	0.072
Yes	37 (5.4%)	29 (8.4%)	–	–
No	642 (94.6%)	318 (91.6%)	–	–
Family history (%)	–	–	2.16	0.142
Yes	61 (9.0%)	22 (6.3%)	–	–
No	618 (91.0%)	325 (93.7%)	–	–
Neurological conditions (%)	–	–	0.33	0.565
Normal	466 (68.6%)	232 (66.9%)	–	–
Abnormal	213 (31.4%)	115 (33.1%)	–	–
Multiple seizure forms			0.23	0.633
Yes	163 (24.0%)	88 (25.4%)		
No	516 (76.0%)	259 (74.6%)		
Outcome (%)	–	–	0.01	0.944
DRE	229 (33.7%)	118 (34.0%)	–	–
DSE	450 (66.3%)	229 (66.0%)	–	–

SE, status epilepticus; DEE, development and epileptic encephalopathy; EEG, electroencephalogram; DRE, drug-resistant epilepsy; DSE, drug-sensitive epilepsy.

Categorical variables were compared by Chi-square (χ^2^) test.

### Model establishment

**Figure 2:**
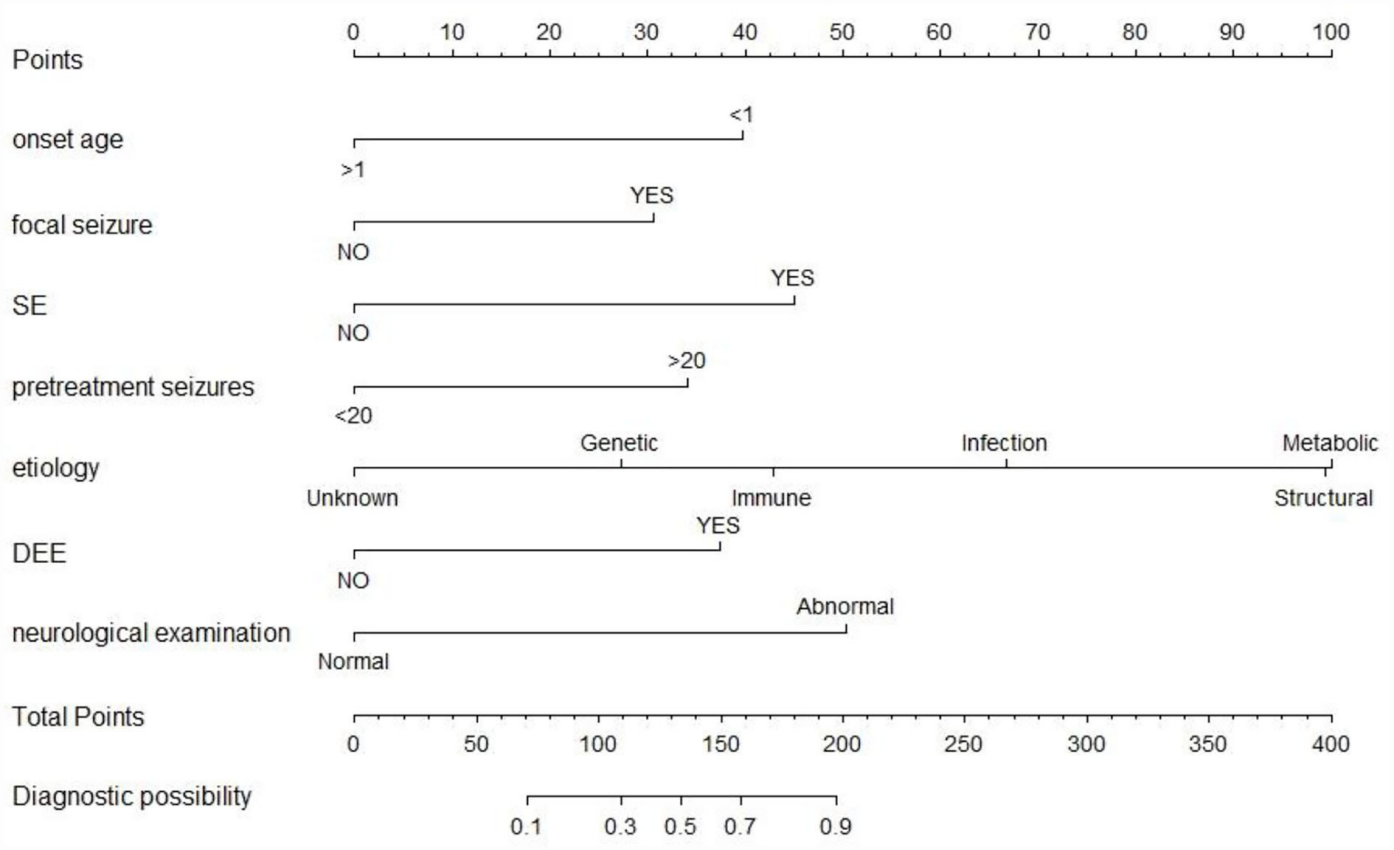
The nomogram prediction model of individual DRE. SE, status epilepticus; DEE, development and epileptic encephalopathy. The top row is used for point assignments for each variable. Rows 2–8 indicated the variables included in the nomogram. The sum of the points is located on the total points line. The lowest row showed the diagnostic possibility of DRE.

**Figure 3:**
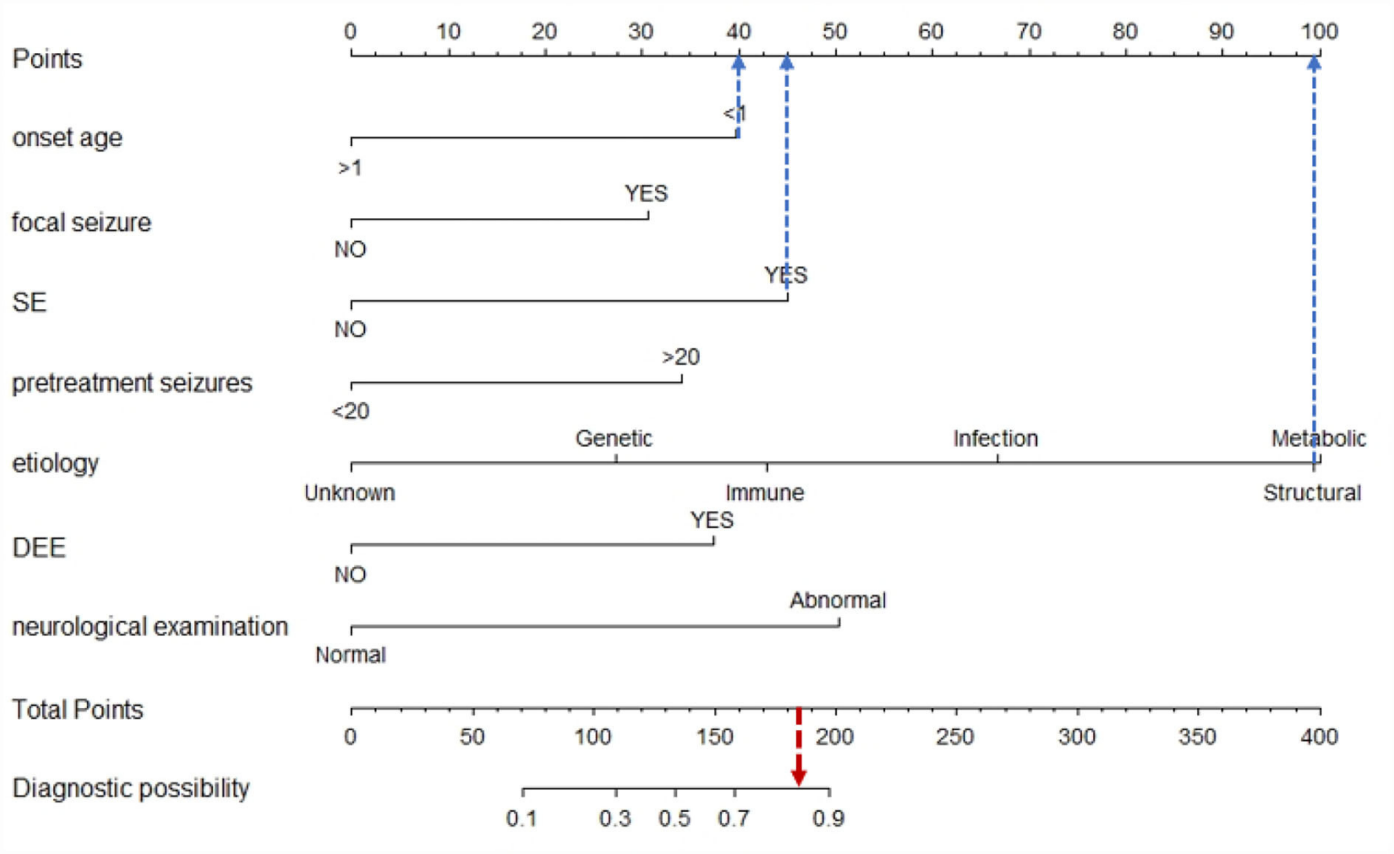
Example of the nomogram prediction model for individual DRE. If there was an epileptic child aged 6 months old, with SE, structural etiology, 10 pre-treatment seizures, but without focal seizure, DEE, or neurological abnormalities, then the cumulative score of predicting factors was 40 + 45 + 99 + 0 + 0 + 0 + 0 = 184, and the corresponding likelihood for DRE was 0.84 (84%), indicating that this child was at 84% possibility for DRE.

Univariate analysis showed that onset age <1, status epilepticus (SE), focal seizure, clustered seizures, >20 pre-treatment seizures, radiological abnormalities, clear etiology (caused by genetic, structural, metabolic, or infectious), DEE, abnormalities of initial EEG, perinatal asphyxia, neurological abnormalities, and multiple seizure forms were risk factors for DRE with statistical significances. However, gender, family history, and history of febrile convulsions were not associated with DRE (Table 2). Based on the collinearity diagnosis of 12 risk factors, we found the variance inflation factor (VIF) was <2, the tolerance was >0.1, the eigenvalue was close to 0, and the conditional indexes were <30. Thus, no collinearity in these risk variables could be deduced (Table 3 and Supplementary Table 1). Children with radiological abnormalities, moreover, are frequently characterized by symptomatic epilepsy, and children with clustered seizures are more prone to have >20 pre-treatment seizures. Taking this into consideration, the risk factors radiological abnormalities and clustered seizures were excluded in this study. The risk factors for DRE with statistical significance in the univariate analysis were further analyzed with unconditional binary multifactor logistic regression, and the result showed that onset age <1, SE, focal seizure, >20 pre-treatment seizures, clear etiology (caused by genetic, structural, metabolic or infectious), DEE, and neurological abnormalities were independent risk factors for DRE (Table 2). Therefore, seven independent risk factors were adopted and analyzed in the nomogram for predicting the incidence of individual DRE (Figure 2). For example, if there was an epileptic child aged 6 months old, with SE, structural etiology, 10 pre-treatment seizures, but without focal seizure, DEE, or neurological abnormalities, then the cumulative score of predicting factors was 40 + 45 + 99 + 0 + 0 + 0 + 0 = 184, and the corresponding likelihood for DRE was 0.84 (84%), indicating that this child was at 84% possibility for DRE (Figure 3).

**Table 2:** Univariate and multivariate logistic regression in the training set.

	**Univariate analysis**		**Multivariate analysis**	
	**OR (95%CI)**	***P* value**	**OR (95%CI)**	***P* value**
Gender (male)	0.82 (0.59–1.13)	0.219	NA	NA
Onset age <1	3.57 (2.45–5.18)	<0.001*	3.96 (2.24–6.98)	<0.001*
SE	5.32 (3.16–8.97)	<0.001*	4.76 (2.21–10.28)	<0.001*
Focal seizure	3.51 (2.49–4.95)	<0.001*	2.88 (1.76–4.73)	<0.001*
Clustered seizures	7.16 (5.00–10.26)	<0.001*	NA	NA
>20 pretreatment seizures	4.33 (3.09–6.07)	<0.001*	3.25 (1.99–5.29)	<0.001*
Radiological abnormalities	30.30 (18.43–49.82)	<0.001*	NA	NA
Etiology		<0.001*		<0.001*
Genetic	–	–	–	–
Structural	8.76 (4.19–18.32)	<0.001*	12.16 (4.69–31.55)	<0.001*
Metabolic	6.10 (2.39–15.52)	<0.001*	12.41 (3.78–40.74)	<0.001*
Immune	1.24 (0.43–3.52)	0.691	1.72 (0.46–6.39)	0.421
Infectious	3.45 (1.88–6.34)	<0.001*	3.92 (1.73–8.85)	0.001*
Unknown	0.28 (0.16–0.50)	<0.001*	0.39 (0.18–0.83)	0.014*
DEE	4.04 (2.83–5.77)	<0.001*	3.64 (2.13–6.22)	<0.001*
Abnormalities on initial EEG	1.80 (1.05–3.09)	0.033*	NA	NA
Perinatal asphyxia	2.66 (1.53–4.63)	0.001*	NA	NA
History of febrile convulsion	1.54 (0.79–3.00)	0.211	NA	NA
Family history	1.31 (0.76–2.24)	0.332	NA	NA
Neurological abnormalities	5.44 (3.83–7.74)	<0.001*	5.72 (3.45–9.49)	<0.001*
Multiple seizure forms	2.30 (1.60–3.29)	<0.001*	NA	NA

SE, status epilepticus; DEE, development and epileptic encephalopathy; EEG, electroencephalogram.

* *P* <0.05; OR, odds ratio; NA, not consolidation; CI, confidence interval.

**Table 3:** Variance inflation factor (VIF) and tolerance analysis of variables.

**Variables**	***P* value**	**Tol**	**VIF**
Onset age <1	0.000	0.93	1.08
SE	0.000	0.94	1.07
Focal seizure	0.000	0.91	1.11
Clustered seizures	0.000	0.84	1.20
> 20 Pretreatment seizures	0.000	0.85	1.18
Radiographic abnormalities	0.000	0.60	1.67
Etiology	0.910	0.68	1.48
DEE	0.000	0.92	1.09
Abnormalities on initial EEG	0.358	0.97	1.03
Perinatal asphyxia	0.060	0.96	1.04
Neurological abnormalities	0.000	0.90	1.11
Multiple seizure forms	0.053	0.94	1.06

VIF, variance inflation factor; Tol, tolerance; SE, status epilepticus; DEE, development and epileptic encephalopathy; EEG, electroencephalogram.

The (VIF) was <2, the tolerance was >0.1 inferred that there was no collinearity in these risk factors.

### Model validation

**Figure 4:**
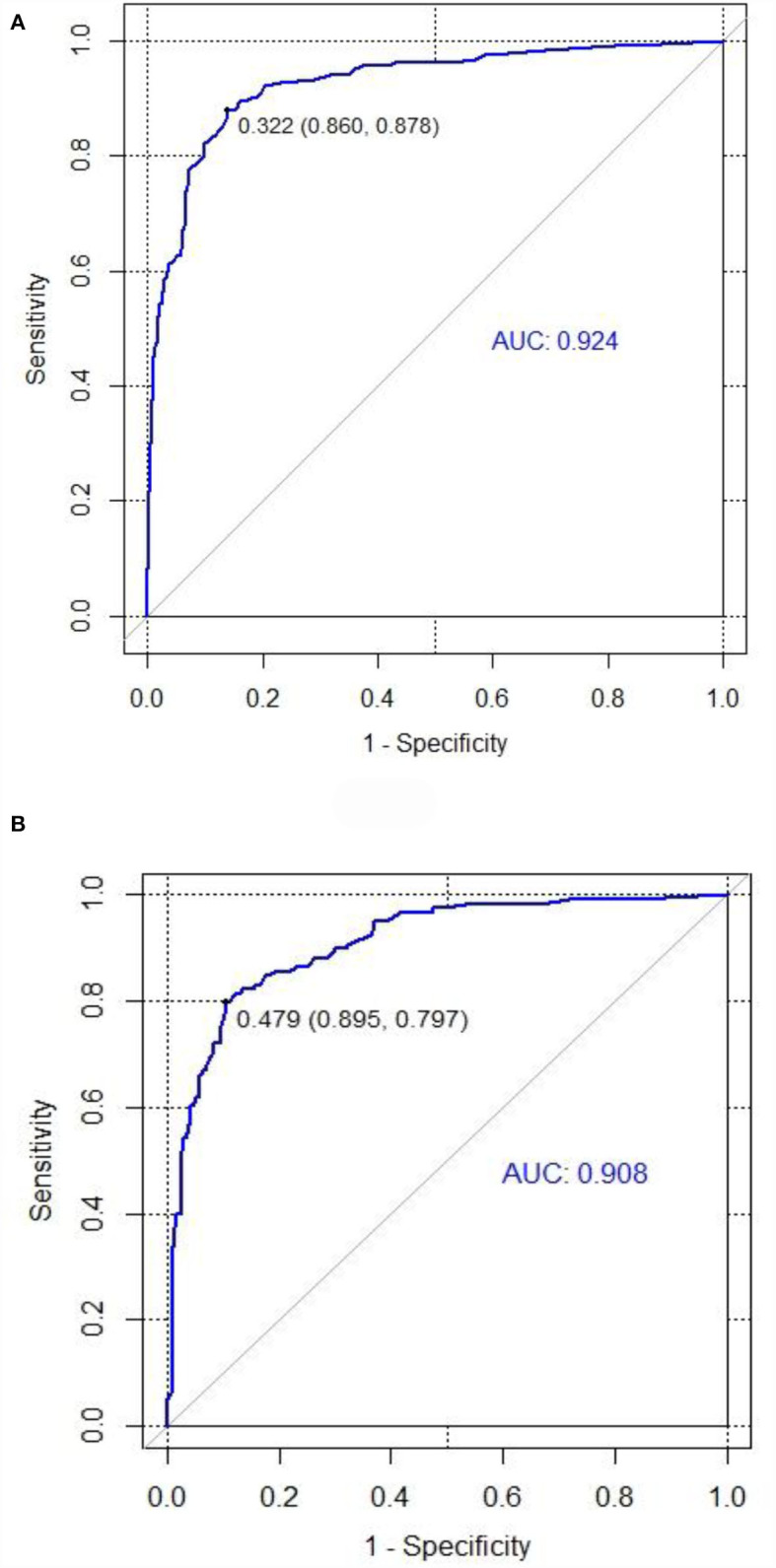
ROC curves for training and validation sets in the nomogram prediction model. **(A)** Training set. **(B)** Validation set (AUC = 0.92, AUC = 0.91). The area under the ROC curve was 0.92 and 0.91 in the training set and the validation set, respectively.

We tested the nomogram's performance through discrimination and calibration. The ROC curve for DRE prediction was drawn by R software. The AUC value was 0.92 for the training set compared to 0.91 for the validation set. The AUC of the nomogram model for both sets was >0.75, suggesting good discrimination for the nomogram prediction model (Table 4, Figure 4).

**Table 4:** AUCs of ROC curves for the nomogram and variables from the logistic regression model in the training as well as validation sets.

	**Training set**			**Validation set**		
	**AUC**	**95%CI**	***P* value**	**AUC**	**95%CI**	***P* value**
Nomogram	0.92	0.92–0.93	<0.001	0.91	0.90–0.91	<0.001
Onset age	0.62	0.58–0.66	<0.001	NA		
>20 pretreatment seizures	0.67	0.63–0.72	<0.001	NA		
Etiology	0.74	0.71–0.78	<0.001	NA		
DEE	0.64	0.60–0.69	<0.001	NA		
Neurological abnormalities	0.69	0.64–0.73	<0.001	NA		
SE	0.59	0.54–0.63	<0.001	NA		
Focal seizure	0.65	0.61–0.69	<0.001	NA		

AUC, area under the curve; ROC, receiver operating characteristic; SE, status epilepticus; DEE, development and epileptic encephalopathy; NA, not consolidation; CI, confidence interval.

**Figure 5:**
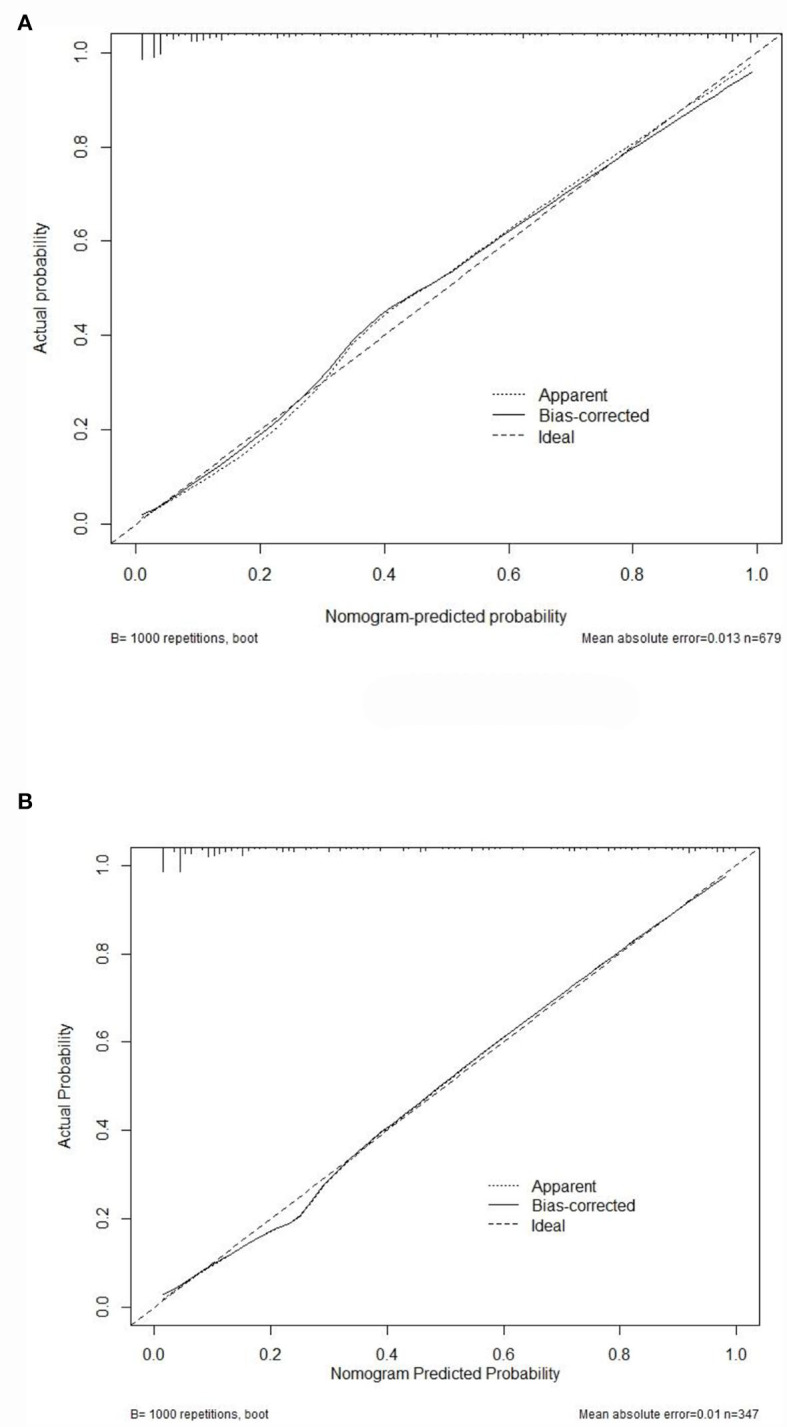
The calibration curves for the nomogram. Calibration plots of predictive DRE risk in the training set **(A)** as well as the external validation set **(B)**. The x-axis showed the nomogram-predicted probability, while the y-axis showed the actual probability of DRE. Perfect prediction would correspond to the 45° dashed line.

**Figure 6:**
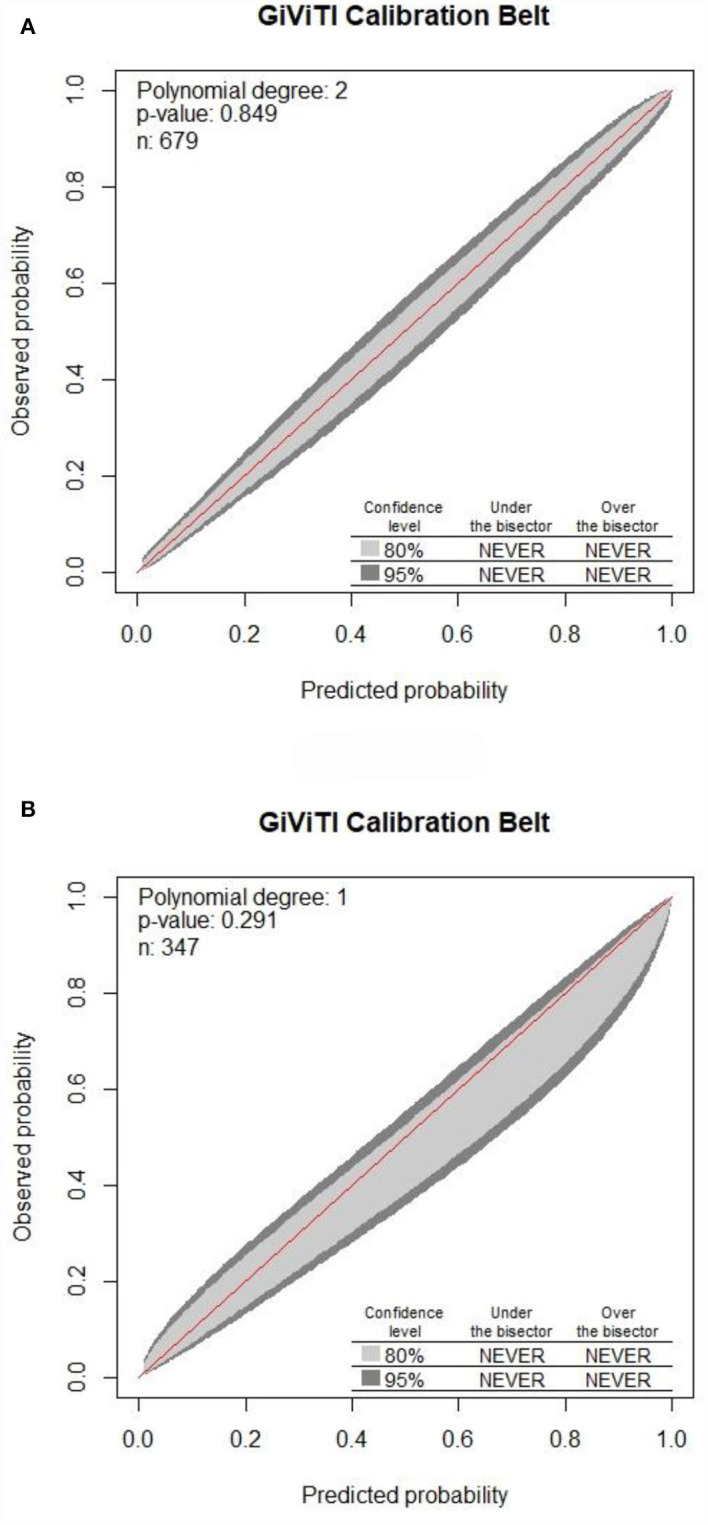
Calibration of the nomogram for DRE prediction in the training set along with the validation set. The 80% CI and 95% CI of the GiViTI calibration belt for either the training set **(A)** or validation set **(B)** did not surpass the diagonal line, with *P*-values of 0.849 for the training set compared to that of 0.291 for the validation set in the GiViTI calibration belt (*P* > 0.05).

Internal validation of the nomogram was conducted through bootstrap analyses with 1,000 resamples. The C-index was 0.92 (95% CI 0.92–0.93) and 0.91 (95% CI 0.90–0.91) in the training and validation sets, respectively. Based on the two calibration curves, a good agreement was demonstrated between the predicted and observed values for DRE (Figure 5). The 80% and 95% CI of the GiViTI calibration belt did not cross the diagonal line, presenting *P*-values of 0.849 and 0.291 for the training set and for the validation set, respectively (*P* > 0.05), with no statistical significance (Figure 6). We also calculated the accuracy rate for the nomogram in each validation set: the internal validation (accuracy: 0.87, 95% CI 0.88–0.90, sensitivity:0.93, specitivity:0.76) and the external validation (accuracy:0.87, 95%, CI 0.84–0.90, sensitivty:0.93, specificity: 0.76). These findings indicated that the validation set had strong consistency with the training set. Thus, the nomogram prediction model was perfectly calibrated.

## Discussion

Epilepsy is a brain disease persistently prone to result in epileptic seizures (28). The incidence of epilepsy in children was 144/100 000 and 58/100 000 person-years for those aged <1 year and 1–10 years, respectively. The cumulative incidence of epilepsy reached up to 0.66% for the children at 10 years old, among which 0.62% with active epilepsy (29). DRE accounts for 20–30% of all patients with epilepsy (3–5). As reported, there are 13 million disability-adjusted life years due to epilepsy each year globally (30). Long-term recurrent seizures exert a serious influence on the physical/mental health of children, as well as their quality of life. If the children at high risk of DRE could be effectively identified at the early stage, personalized interventions could be adopted to prevent its occurrence, reduce severe comorbidities, and improve prognosis. The risk factors for DRE currently reported have been inconsistent, if not controversial. Furthermore, most prediction models lack high replicability or sufficient evidence, and risk variables were not quantified specifically. Based on the above, it is of great significance to explore the risk factors for DRE among epileptic children and establish an easy-to-operate visual scale for the assessment of therapeutic efficacy. In our study, the established nomogram prediction model was found to be capable of identifying patients with DRE at diagnosis before the treatments, and the results suggested that this prediction model was able to identify patients with high-risk DRE at least 1 year earlier than the current practice that waits for failing from two AEDs.

### Risk factors for DRE

In this study, compared to the 450 children with DSE, the independent risk factors for 229 children with DRE included onset age <1, SE, focal seizure, >20 pre-treatment seizures, clear etiology (caused by genetic, structural, metabolic, or infectious), DEE, and neurological abnormalities. Specifically, the risk of onset age < 1 year old in children with DRE was 3.96 times of those with onset age >1, which was consistent with other published literature (3). Infancy is the most critical period in brain development. Brain maturity may be related to the high incidence of seizures in infancy (31–33). Besides, the frequency of focal epilepsy in children with DRE was 2.88 times that of children with DSE in this study. As common focal epilepsy, hippocampal sclerosis has been recognized as the most common pathology contributing to drug-resistant mesial temporal lobe epilepsy (34), while patients with focal frontal epilepsy (35, 36) are prone to be drug-resistant. Thus, it can be inferred that patients with focal origins are more inclined to develop DRE. Additionally, we found that more than 20 pre-treatment seizures were an independent risk factor for DRE, which was consistent with the results of a 30-year longitudinal cohort study (37).

Regarding the etiology of epilepsy, epilepsy may be classified into structural, infectious, genetic, metabolic, immune, or unknown subgroups according to the suggestions of ILAE (22). After a series of auxiliary examinations, we were able to discover a clear and definitive etiology for the disease in the majority of the children with DRE, and some patients may have two or more reasons. When compared to those with an unclear etiology, epilepsy with a definite etiology was harder to treat and was more inclined to progress to DRE. Autoimmune encephalitis can cause epilepsy; however, it is not the risk factor of DRE. The prognosis of autoimmune encephalitis is correlated with the location of antigen and antibody, which can be divided into anti-neuron cell surface antibody and anti-neuronal intracellular antibody, the former is more common, and has a low probability of progressing to DRE, but the latter easily progress to DRE. In a word, anti-neuron cell surface antibody, the most common type of autoimmune encephalitis, is less likely to develop into DRE (38). The prognosis was poorest in patients with structural etiology, followed by metabolic, infectious, and genetic etiology, respectively; this differed from the results of previous studies based on the classification of idiopathic, symptomatic, and cryptogenic epilepsy (39, 40).

There are four types of drug resistance in clinical practice: de novo continuous drug resistance, reversal of drug resistance, possibly in an intermittent pattern in which periods of remission are followed by periods of uncontrolled seizures, and progression to drug resistance of delayed onset with persistent loss of efficacy after the control initially (41). Therefore, the condition of DRE may be recognized in more than 50% of early-stage patients. Focal signs implicating or localizing cerebral pathology are identified through the neurological examination (42), where dysmorphic features can suggest genetic syndromes (7). For instance, increased tone on one side of the body could suggest lesions in the contralateral hemisphere, such as cortical dysplasia (42). Stigmata of neurocutaneous syndromes are constituted by the facial port-wine stain of Sturge–Weber syndrome; café au lait spots and iris hamartomas of neurofibromatosis; and facial angiofibroma, periungual fibromas, hypomelanotic macules, as well as shagreen patches of tuberous sclerosis complex (TSC) (43). In our study, it was found that the risk factor of neurological abnormalities was an independent predictor for DRE, with its frequency in children with DRE5.72 times that of children with DSE. Multiple studies have proved the strong correlation between MRI abnormalities and the incidence of DRE (44–46), which was consistent with the result of our study. In our study, it was found that the risk of abnormalities on head MRI in children with DRE was 30.30 times higher than that in children with DSE (*P* < 0.001, OR = 30.30, 95% CI 18.43–49.82). However, as an auxiliary investigation, abnormalities on MRI were found to be collinear with the clinical etiology of DRE and were thus excluded by multiple logistic regression. Etiology was the biggest risk factor for DRE, according to the odds ratios in this study, followed by neurological abnormalities, SE, onset age <1, DEE, >20 pre-treatment seizures, and focal origins, respectively.

### Establishment of a prediction model

The establishment of prediction models is conducive to the application of scoring tools in clinical guidelines, benefits doctor–patient communication, as well as provides an optimal allocation of healthcare resources. The essence of prediction models is to explore the law between independent and dependent variables through statistical analysis and to quantitate the relationship between predictive factors and outcomes. So far, prediction models mainly included formulas, scoring tables, web calculators, mobile APPs, nomograms, and so on. Nomogram achieves data visualization based on a complex mathematical formula. It makes use of the variables which are independent risk factors to picture a prediction model which is capable of quantifying a specific event's probability in clinical practice. After being listed separately, each variable is assigned a point, which is a weighted number based on the regression coefficient. Subsequently, the score for all the variables is summed up, followed by the matching to a scale to predict the outcomes. The formula can also be alternatively programming into a calculator based on the computer or smartphone. Once the variables are entered, the possibility of a specific event would be calculated (47). In other words, nomogram has the potential of transforming complicated regression equations into the visual figure, making prediction models easy-to-operate and allowing clinicians to conveniently obtain the likelihood of diseases. In recent years, nomogram has been an influencing element in healthcare decision-making at a system level. A well-established nomogram designed to solve a practical and confusing problem, if correctly interpreted or used, will be beneficial for clinicians, as well as patients. In this study, nomograms were drawn using R software (rms package) on the basis of the regression coefficients of independent risk factors for children with DRE. Throughout the establishment and validation of the model, we stand by the transparent reporting of a multivariable prediction model for individual prognosis or diagnosis (TRIPOD) statement (48, 49). Because the nomogram can generate a numerical probability for individual DRE by integrating various independent and predictive risk factors, it fits our need model that combines demographic, genetic, biological, and clinical data. The clinicians no longer need to memorize complicated formulas. Nomograms can be beneficial for clinicians and patients not only in planning personalized treatments but also in providing a reference for decision-making. As far as we know, our nomogram model is very innovative for DRE prediction, since no relative visual tool has been established before.

### Discrimination assessment of the nomogram prediction model

ROC curves have been widely used in the fields of medicine and machine learning since they are the most convenient evaluation tool for models. AUC was adopted to assess each predictor and how the model discriminates the patients with DRE from DSE. The value of AUC is associated with the model's discrimination ability. Generally speaking, the AUC between 0.5 and 0.75 means a poor ability, while AUC > 0.75 suggests a good ability (50). In this study, the AUC was 0.92 for the training set compared to 0.91 for the validation set, both larger than 0.75, suggesting a good discrimination ability for the nomogram prediction model.

### Calibration assessment of the nomogram prediction model

The nomogram's performance was assessed by concordance (C-index), calibration curves along with GiViTI calibration belts. C-index exceeded 0.90 in all sets indicating good consistency of the nomogram. A prediction model is considered to be well-calibrated when it predicts the clinical event accurately. *P*-value of the GiViTI calibration belt < 0.05 indicates a not-so-perfect model. In this study, the 80% CI and 95% CI of the GiViTI calibration belt did not cross the dashed line (diagonal line), with *P*-values of 0.849 for the training set compared to that of 0.291 for the validation set in the GiViTI calibration belt (*P* > 0.05), indicating that the predicted risks had strong consistency with the observed outcomes, suggesting the perfect calibration of the prediction model.

### Limitations of this study

This study also has shortcomings. To begin with, as this is a retrospective study, there may be a recall bias and, obviously, a selection bias. We used a strict inclusion criterion and collected sufficient samples to reduce bias. According to the results of PASS, the calculated sample size for the DSE and DRE groups was 186 children in total. However, the children included in this study were far more than the calculated minimum sample size. Second, the population for the model establishment came from a single center. Although we have applied external validations, the external validation was based on two hospitals; thus, data from other centers for more external validations are still necessary. Therefore, we are expecting other institutions to join this research and strengthen the prediction model in future.

## Conclusion

1. The clinical information of children with DRE and DSE was retrospectively analyzed. The nomogram prediction model of individual DRE was constructed through univariate and multivariate logistic analyses.
2. Onset age < 1 year old, SE, focal seizure, >20 pre-treatment seizures, clear etiology (caused by genetic, structural, metabolic, or infectious), DEE, and neurological abnormalities were all independent risk factors for DRE.
3. Nomogram realized the visualization of the prediction model for DRE. Moreover, the model was well discriminated and calibrated among children, for model development and model validation. Based on the nomogram for DRE, we would have the potential not only to accurately predict the risk of individual DRE but also in achieving an early identification of DRE children. The predicted probability was strongly consistent with the real situation.

## Data availability statement

The original contributions presented in the study are included in the article/Supplementary material, further inquiries can be directed to the corresponding author/s.

## Ethics statement

Written informed consent was obtained from the individual(s), and minor(s)' legal guardian/next of kin, for the publication of any potentially identifiable images or data included in this article.

## Author contributions

HG drafted the manuscript and contributed to the data acquisition. XC contributed to the conception and design of the study and critically revised and gave final approval for the publication of the article. Both authors contributed to the article and approved the submitted version.

## Funding

This work was granted by the National Natural Science Foundation of China (82171441), Natural Science Foundation of Jiangsu Province (BK20201175), and Yancheng Science Development Project (YK2021006).

## Conflict of interest

The authors declare that the research was conducted in the absence of any commercial or financial relationships that could be construed as a potential conflict of interest.

## Publisher's note

All claims expressed in this article are solely those of the authors and do not necessarily represent those of their affiliated organizations, or those of the publisher, the editors and the reviewers. Any product that may be evaluated in this article, or claim that may be made by its manufacturer, is not guaranteed or endorsed by the publisher.
