# Supplementary material for: Development and validation of a nomogram for the early prediction of drug resistance in children with epilepsy
Source: Front Pediatr. 2022 Aug 30;10:905177. doi: 10.3389/fped.2022.905177 (PMC9468368; doi:10.3389/fped.2022.905177)
Supplement: Supplementary file 1 [file Table_1.pdf]

Supplementary Table 1 Analysis of eigenvalue, conditional index, variance proportions

**Eigenvalue**	**Conditional index**	**Variance proportions**											
		Onset age<1	SE	Focal seizure	Clustered seizures	pretreatment seizures	Radiographic abnormalities	Etiology	Neurological abnormalities	Multiple seizure forms	Perinatal asphyxia	DEE	Abnormalities on initial EEG
6.32	1.00	0.01	0.00	0.01	0.01	0.01	0.00	0.00	0.01	0.01	0.00	0.01	0.00
1.02	2.49	0.04	0.15	0.01	0.00	0.00	0.06	0.01	0.00	0.01	0.31	0.01	0.01
0.89	2.66	0.03	0.29	0.01	0.00	0.00	0.04	0.00	0.00	0.08	0.36	0.00	0.00
0.81	2.79	0.01	0.21	0.00	0.03	0.02	0.07	0.01	0.09	0.12	0.13	0.09	0.00
0.72	2.95	0.31	0.14	0.00	0.00	0.00	0.03	0.00	0.01	0.27	0.00	0.17	0.00
0.67	3.06	0.49	0.11	0.01	0.00	0.00	0.04	0.00	0.08	0.13	0.18	0.02	0.00
0.61	3.23	0.02	0.02	0.04	0.02	0.13	0.01	0.00	0.34	0.15	0.00	0.29	0.00
0.57	3.32	0.02	0.04	0.02	0.14	0.32	0.01	0.00	0.01	0.08	0.00	0.39	0.00
0.52	3.50	0.03	0.03	0.10	0.01	0.01	0.26	0.00	0.42	0.14	0.00	0.02	0.01
0.39	4.04	0.02	0.01	0.01	0.74	0.48	0.00	0.00	0.02	0.00	0.00	0.00	0.00
0.34	4.30	0.01	0.00	0.79	0.05	0.01	0.16	0.01	0.00	0.01	0.01	0.01	0.03
0.10	7.83	0.01	0.00	0.00	0.00	0.01	0.15	0.33	0.02	0.00	0.01	0.00	0.67
0.03	13.82	0.01	0.00	0.00	0.00	0.00	0.17	0.63	0.00	0.00	0.01	0.00	0.28

Eigenvalue close to 0， Conditional index＜30， inferred that there was no collinearity in these risk factors

Supplementary Table 2 Antiepileptic drugs usage and effective blood concentration in children

AED	Abbreviation	maintenance dose	Effective blood concentration
**Valproic acid**	VPA	20-30mg/kg/day	50-100ug/ml
**Topiramate**	TPM	3-6mg/kg/day	5-20ug/ml
**Levetiracetam**	LEV	20-60mg/kg/day	20-40ug/ml
**Oxcarbazepine**	OXC	20-30mg/kg/day	10-35ug/ml
**Lacosamide**	LAC		
11-30kg		6-12mg/kg/day	
30-50kg		4-8mg/kg/day	
＞50kg		100mg/day	
**Zonisamide**	ZNS	4-8 mg/kg/day	
**Carbamazepine**	CBZ	10-20mg/kg/day	4-12mg/L
**Clonazepam**	CZP	0.1-0.2mg/kg/day	20-90ug/L
**Phenobarbital**	PB	3-5mg/kg/day	10-40mg/L
**Phenytoin**	PHT	4-8mg/kg/day	10-20ug/ml
**Lamotrigine**	LTG		3-14ug/ml
monotherapy		2-10mg/kg/day	
Combined with enzyme-inducing AEDs		5-15mg/kg/day	
Combined with Valproate		1-5mg/kg/day	
**Vigabatrin**	VGB	80-100mg/kg/day	
